# Admission lactate level and the GRACE 2.0 score are independent and additive predictors of 30-day mortality of STEMI patients treated with primary PCI—Results of a real-world registry

**DOI:** 10.1371/journal.pone.0277785

**Published:** 2022-11-16

**Authors:** Dominika Szabo, Andras Szabo, Levente Magyar, Gyongyver Banhegyi, Szilvia Kugler, Anita Pinter, Vencel Juhasz, Mihaly Ruppert, Attila Olah, Zoltan Ruzsa, Istvan Ferenc Edes, Andrea Szekely, David Becker, Bela Merkely, Istvan Hizoh

**Affiliations:** 1 Heart and Vascular Center, Semmelweis University, Budapest, Hungary; 2 School of PhD Studies, Semmelweis University, Budapest, Hungary; 3 Independent Researcher, Budapest, Hungary; 4 Division of Invasive Cardiology, 2^nd^ Department of Internal Medicine, University of Szeged, Szeged, Hungary; 5 Department of Oxiology and Emergency Care, Semmelweis University, Budapest, Hungary; University of Florida, UNITED STATES

## Abstract

**Background:**

In many of the risk estimation algorithms for patients with ST-elevation myocardial infarction (STEMI), heart rate and systolic blood pressure are key predictors. Yet, these parameters may also be altered by the applied medical treatment / circulatory support without concomitant improvement in microcirculation. Therefore, we aimed to investigate whether venous lactate level, a well-known marker of microcirculatory failure, may have an added prognostic value on top of the conventional variables of the “Global Registry of Acute Coronary Events” (GRACE) 2.0 model for predicting 30-day all-cause mortality of STEMI patients treated with primary percutaneous coronary intervention (PCI).

**Methods:**

In a prospective single-center registry study conducted from May 2020 through April 2021, we analyzed data of 323 cases. Venous blood gas analysis was performed in all patients at admission. Nested logistic regression models were built using the GRACE 2.0 score alone (base model) and with the addition of venous lactate level (expanded model) with 30-day all-cause mortality as primary outcome measure. Difference in model performance was analyzed by the likelihood ratio (LR) test and the integrated discrimination improvement (IDI). Independence of the predictors was evaluated by the variance inflation factor (VIF). Discrimination and calibration was characterized by the c-statistic and calibration intercept / slope, respectively.

**Results:**

Addition of lactate level to the GRACE 2.0 score improved the predictions of 30-day mortality significantly as assessed by both LR test (LR Chi-square = 8.7967, p = 0.0030) and IDI (IDI = 0.0685, p = 0.0402), suggesting that the expanded model may have better predictive ability than the GRACE 2.0 score. Furthermore, the VIF was 1.1203, indicating that the measured lactate values were independent of the calculated GRACE 2.0 scores.

**Conclusions:**

Our results suggest that admission venous lactate level and the GRACE 2.0 score may be independent and additive predictors of 30-day all-cause mortality of STEMI patients treated with primary PCI.

## Introduction

Mortality risk assessment is an integral part of the daily clinical practice. Individual mortality risk can be estimated by scoring algorithms. These may provide useful information for patients / relatives and help physicians to allocate hospital resources. They can be used for risk adjustment for intra-organizational quality monitoring and for inter-organizational comparisons of health care providers with different case mixes. Thus, they may contribute to an improved quality of care. Furthermore, risk models may be helpful in clinical trial design identifying patients with the needed risk profile thereby increasing statistical power or reducing sample size and costs [1].

According to the current guidelines, the “Global Registry of Acute Coronary Events” (GRACE) 2.0 score is recommended for risk assessment of patients with acute coronary syndrome [2–4]. Also, 30-day risk-adjusted mortality is one of the quality indicators that is used to evaluate quality of care of STEMI patients [3]. As in many of the current risk estimation algorithms, heart rate and systolic blood pressure are key predictors of the GRACE 2.0 score [1]. Nevertheless, these parameters may also be altered by the applied medical treatment / circulatory support without concomitant improvement in microcirculatory failure / prognosis of the patient. Since if oxygen delivery is inappropriate and compensatory mechanisms are exhausted, global tissue hypoxia develops with anaerobic metabolism and subsequent lactate production [5], we aimed to investigate whether venous lactate level, a marker of microcirculatory failure, may have an added prognostic value on top of the extensively validated GRACE 2.0 score for predicting 30-day all-cause mortality of STEMI patients treated with primary percutaneous coronary intervention (PCI).

## Methods

### Study design, outcome measures

**Fig 1:**
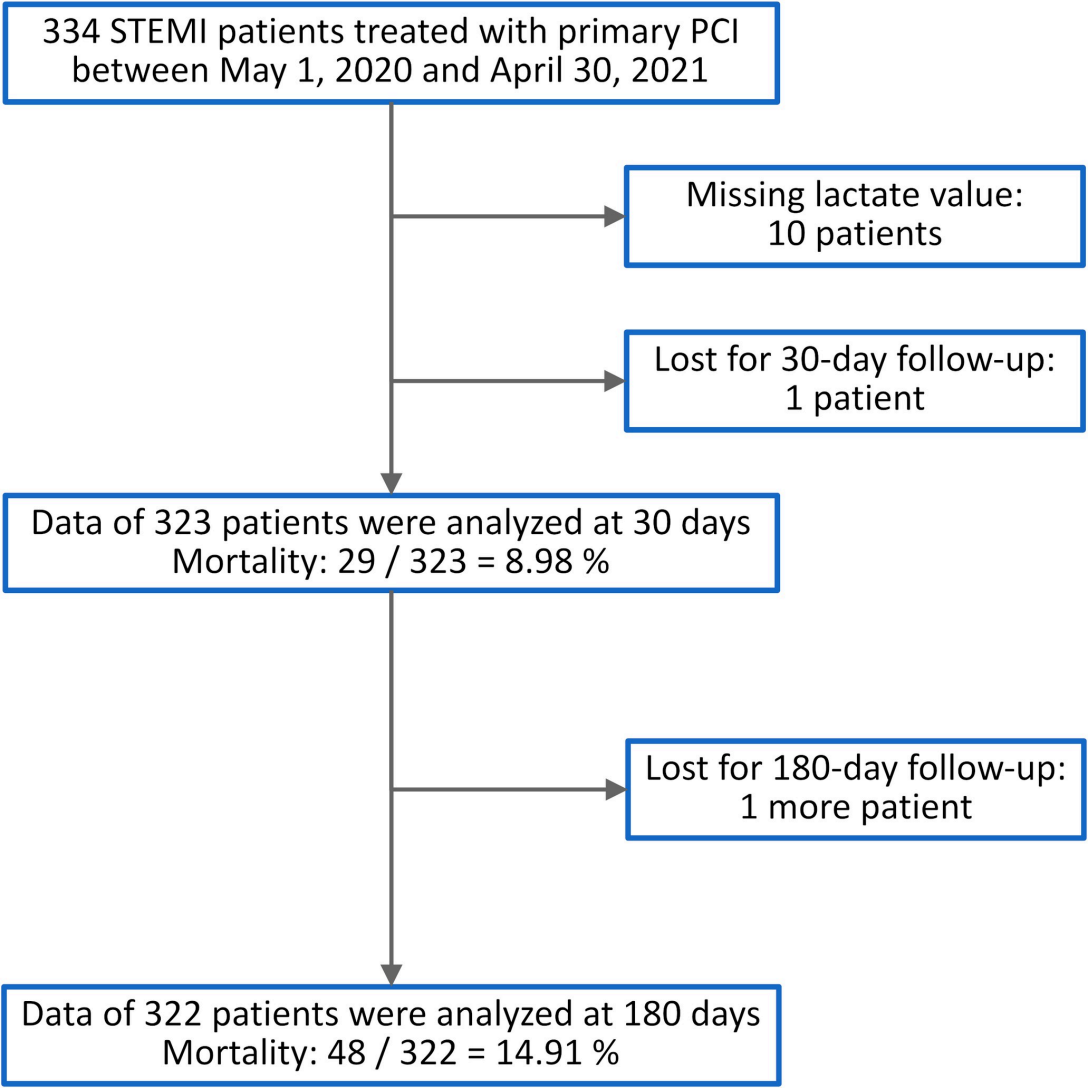
Patient flow chart. For details: see text. PCI: percutaneous coronary intervention; STEMI: ST-segment elevation myocardial infarction.

In a pilot real-world prospective single-center registry, data of 334 STEMI cases were collected from May 2020 through April 2021. All patients were treated with primary PCI using standard techniques within 12 hours from symptom onset. All but 10 of them underwent venous blood gas analysis at cardiac care unit admission. One patient was lost for follow-up at 30 days and another one at 180 days (Fig 1). To evaluate the predictive role of venous lactate level, nested logistic regression models were built using the GRACE 2.0 score alone and with the addition of venous lactate with 30-day all-cause mortality as the dependent variable / primary outcome measure. Similarly, in-hospital and 180-day all-cause mortalities were also studied as secondary outcomes of interest by constructing nested logistic and Cox regression models, respectively.

This was an observational study using a single-center registry. The blood sampling for routine laboratory analysis was performed according to the institutional protocol, in that venous blood gas analysis is included (i.e., no specific study-related intervention was done). All patients gave written informed consent to be available for follow-up by means of hospital records, regular follow-up visits, and records of the National Health Insurance Fund. Patient data were prospectively collected in the Heart and Vascular Center of the Semmelweis University, Budapest, Hungary according to applicable laws and regulations: 1997 XLVII Act on the Handling and Protection of Health and Related Personal Data which was modified by Acts CCXLIV of 2013 and CXVIII of 2018 and Decrees 15/2014 and 49/2018 of the Ministry of Human Resources of Hungary. All Hungarian health care providers are obliged by the above-mentioned laws to provide anonymized data of all patients with myocardial infarction for the prospective National Registry of Myocardial Infarction. The use of these institutional anonymized data for this specific scientific research was approved by the head of the institution and the Regional Ethics Committee on Human Research of the Semmelweis University (approval number: SE RKEB: 4/2021). The study was conducted according to the principles of the Declaration of Helsinki.

### Statistical analysis

Nested logistic regression models were constructed using the 6-month score value of the GRACE 2.0 algorithm alone (base model, online calculator available at https://www.outcomes-umassmed.org/grace/acs_risk2/index.html) and with the addition of venous lactate (expanded model) with 30-day all-cause mortality as the dependent variable / primary outcome measure. As secondary outcomes of interest in-hospital and 180-day all-cause mortalities were also assessed by nested logistic and Cox regression models, respectively. Presence of non-linear relationships of the continuous variables (GRACE 2.0 score and lactate level) to log odds / log relative hazard of the dependent variable were explored using restricted cubic splines which were evaluated graphically and by formal Wald testing for linearity. Model performance was characterized by receiver operating characteristic curve analysis (ROC, c-statistic), calibration intercept and slope, and, in case of logistic regression models, the le Cessie-van Houwelingen-Copas-Hosmer test for global goodness of fit. Difference in model performance was primarily analyzed by the likelihood ratio (LR) test. Though the application of the integrated discrimination improvement (IDI, i.e. the change in the discrimination slope) and the widely used receiver operating characteristic curve analysis (ROC, c-statistic) for model selection has been criticized, they have also been performed [6–8]. The correlated ROC curves were compared by a bootstrap test with 10000 resamples. Independence of the predictors (lack of collinearity) was evaluated by the variance inflation factor (VIF) using a cut-off value of 5. Internal validation of the models’ performance was carried out by bootstrap resampling using 10000 replicates (c-statistic, calibration intercept and slope, mean absolute error) [8]. Moreover, calibration of the apparent and optimism-corrected (overfitting-corrected) models was evaluated graphically by applying a LOWESS (locally weighted scatter plot smoothing) smoother on scatter plots of predicted versus observed probabilities. For better characterization of the studied population, as a quality control measure the observed and expected 30- and 180-day death rates were compared by the exact binomial test. Expected individual 30- and 180-day absolute mortality risks were calculated using the ALPHA [1, 9, 10] and GRACE 2.0 [2] scores, respectively. All statistical analyses and graphical interpretation of the results were carried out with R version 4.2.1 (R Foundation for Statistical Computing, Vienna, Austria) using the fastStat 1.4, fBasics 3042.89.2, ggplot2 3.3.6, gridExtra 2.3, predictABEL 1.2–4, pROC 1.18.0, rms 6.3–0, and survival 3.3–1 additional packages. A two-tailed p value less than 0.05 was considered statistically significant.

## Results

### Patient characteristics

Demographic, clinical, procedural characteristics of the patients and baseline laboratory data are summarized in Table 1. The real-world nature of the population is reflected by the facts that 12.7% of the cases had cardiac arrest on or prior to admission, whereas 11.8% of them were in cardiogenic shock. Also, the contemporariness of the treatment is shown by the high proportion of transradial interventions (94.4%), the almost exclusive use of drug eluting stents (in one patient with ectatic right coronary artery and huge thrombus burden two 7.0 mm bare metal stents wrapped with a polymer mesh were implanted), and the use of guideline-directed discharge medications (Table 2).

**Table 1:** Demographic, clinical, procedural characteristics and baseline laboratory data.

Variable	Median (IQR) or n (%), N = 323
Age (years)	63.0 (53.0 to 73.0)
Sex (Female)	99 (30.7%)
Height (m)	1.71 (1.65 to 1.77)
Weight (kg)	80.0 (70.0 to 92.0)
BMI (kg/m2)	27.6 (24.2. to 31.0)
Known Hypertension	191 (59.1%)
Known Diabetes Mellitus	67 (20.7%)
Newly Diagnosed Diabetes Mellitus	16 (5.0%)
Known Hyperlipidemia	39 (12.1%)
Current Smoker	55 (17.0%)
Known Peripheral Artery Disease	15 (4.6%)
Known Cerebrovascular Disease	27 (8.4%)
Known Congestive Heart Failure	4 (1.2%)
Previous Angina	55 (17.0%)
Previous Myocardial Infarction	46 (14.2%)
Active or Previous Malignancy	22 (6.8%)
Known Chronic Renal Failure	6 (1.9%)
Known Chronic Obstructive Pulmonary Disease	27 (8.4%)
Onset-to-door time (hours)	3.0 (2.0 to 7.0)
ECG Localization of the STEMI	
Anterior / Left Bundle Branch Block	142 (44.0%)
Inferior	143 (44.3%)
Other	38 (11.8%)
Cardiac Arrest on or Prior to Admission	41 (12.7%)
Initial Non-shockable Rhythm	5 (12.2%)
Initial Shockable Rhythm	36 (87.8%)
Heart Rate (1/min)	80.0 (70.0 to 96.5)
Systolic Blood Pressure (mmHg)	135.0 (113.0 to 153.5)
Killip Class	
1	254 (78.6%)
2	28 (8.7%)
3	3 (0.9%)
4	38 (11.8%)
Intra-Aortic Balloon Pump	6 (1.9%)
Venoarterial Extracorporeal Membrane Oxygenation	6 (1.9%)
Mechanical Ventilation	38 (11.8%)
Transradial Primary Percutaneous Coronary Intervention	305 (94.4%)
Access Site Conversion	16 (5.0%)
Vessel Dilated	
Left Anterior Descending	128 (39.6%)
Left Circumflex	28 (8.7%)
Right Coronary	120 (37.2%)
Left main / Multivessel	47 (14.6%)
Bypass Graft	0 (0.0%)
Type of Percutaneous Coronary Intervention	
Drug Eluting Stent	309 (95.7%)
Bare Metal Stent Wrapped With a Polymer Mesh	1 (0.3%)
Drug Eluting Balloon / Plain Old Balloon Angioplasty	11 (3.4%)
Failed Wire Crossing	2 (0.6%)
Thrombus Aspiration	94 (29.1%)
Glycoprotein IIb/IIIa Receptor Inhibitor	97 (30.0%)
Initial TIMI Flow Grade	
0	166 (51.4%)
1	95 (29.4%)
2	56 (17.3%)
3	6 (1.9%)
Final TIMI Flow Grade	
0	4 (1.2%)
1	0 (0.0%)
2	8 (2.5%)
3	311 (96.3%)
Total Stent Length (mm)	33.0 (24.0 to 53.0 mm)
Initial Hemoglobin (g/L)	140.0 (127.0 to 151.0)
Initial Hematocrit (L/L)	0.41 (0.38 to 0.44)
C-Reactive Protein (mg/L)	3.14 (1.56 to 8.54)
Creatinine (μmol/L)	83.0 (70.0 to 102.0)
Cholesterol (mmol/L)	4.9 (4.1 to 5.7)
LDL Cholesterol (mmol/L)	3.47 (2.75 to 4.22)
HDL Cholesterol (mmol/L)	1.07 (0.93 to 1.29)
Triglycerides (mmol/L)	0.90 (0.64 to 1.25)
Cardiac Troponin T (ng/L)	567.0 (235.0 to 2306.0)
Creatine Kinase-MB (U/L)	69.0 (31.0 to 158.0)
Lactate (mmol/L)	2.2 (1.6–3.3)

**Table 2:** Discharge medication.

Drug	Discharge Medication, n (%), N = 304
Acetylsalicylic Acid	302 (99.3%)
Clopidogrel	114 (37.5%)
Prasugrel	171 (56.3%)
Ticagrelor	18 (5.9%)
Vitamin K Antagonist	13 (4.3%)
Direct Oral Anticoagulant	33 (10.9%)
Beta Blocker	271 (89.1%)
Ivabradine	1 (0.3%)
Angiotensin-Converting Enzyme Inhibitor	240 (78.95%)
Angiotensin Receptor Blocker	30 (9.9%)
Mineralocorticoid Receptor Antagonist	38 (12.5%)
Angiotensin Receptor-Neprilysin Inhibitor	0 (0.0%)
Brain Aminopeptidase A Inhibitor / Angiotensin-Converting Enzyme Inhibitor*	12 (3.9%)
Statin	287 (94.4%)
Proton Pump Inhibitor	290 (95.4%)

* Quantum Genomics Firibastat or Ramipril after Acute Myocardial Infarction for Prevention of Left Ventricular Dysfunction (QUORUM) Randomized Clinical Trial.

As a quality control measure, observed and expected 30- and 180-day mortality rates were analyzed. Observed mortality at 30 days was 29/323 = 8.98%, whereas the expected rate (sum of the individual risks / number of patients) was 8.44%, i.e. the risk adjusted mortality was 8.98%/8.44% = 1.06, p = 0.6891. As to the 180-day data, the risk adjusted mortality was 14.91%/14.64% = 1.02, p = 0.8748 (Fig 1).

### Lactate as predictor

**Fig 2:**
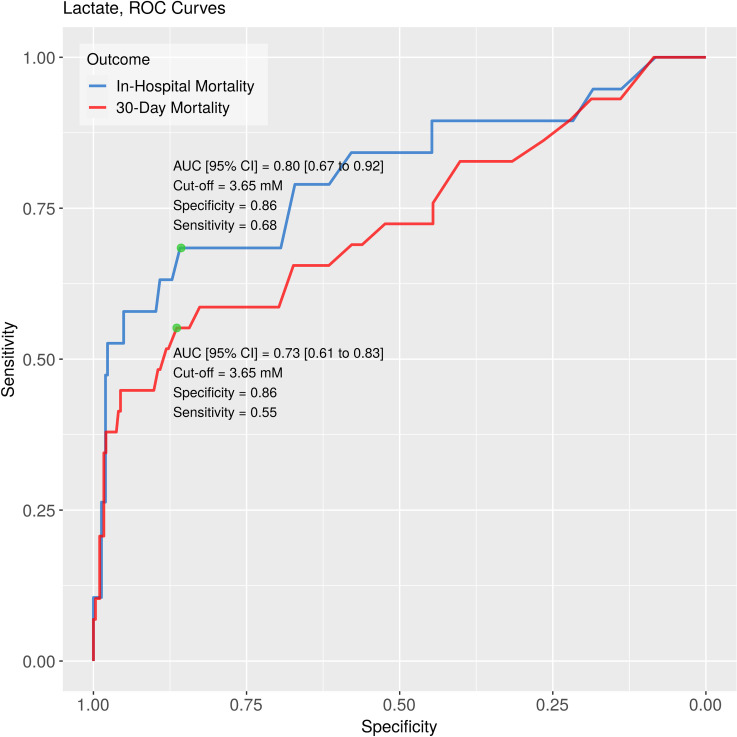
ROC curves for lactate as predictor. Lactate level alone may have good predictive ability for predicting both in-hospital and 30-day mortality. ROC: Receiver Operating Characteristics; AUC: area under curve.

In both logistic regression and Cox modeling with in-hospital, 30-day, and 180-day mortalities as dependent variables venous lactate level proved to be a highly significant predictor (S1 Table). ROC analysis of the lactate level as a single predictor revealed good discriminative ability (Fig 2). According to these analyses, the optimal cut-off point (i.e., the lactate level where the sum of sensitivity and specificity reaches its maximum) for the lactate level was 3.65 mmol/L.

### Primary outcome measure

**Fig 3:**
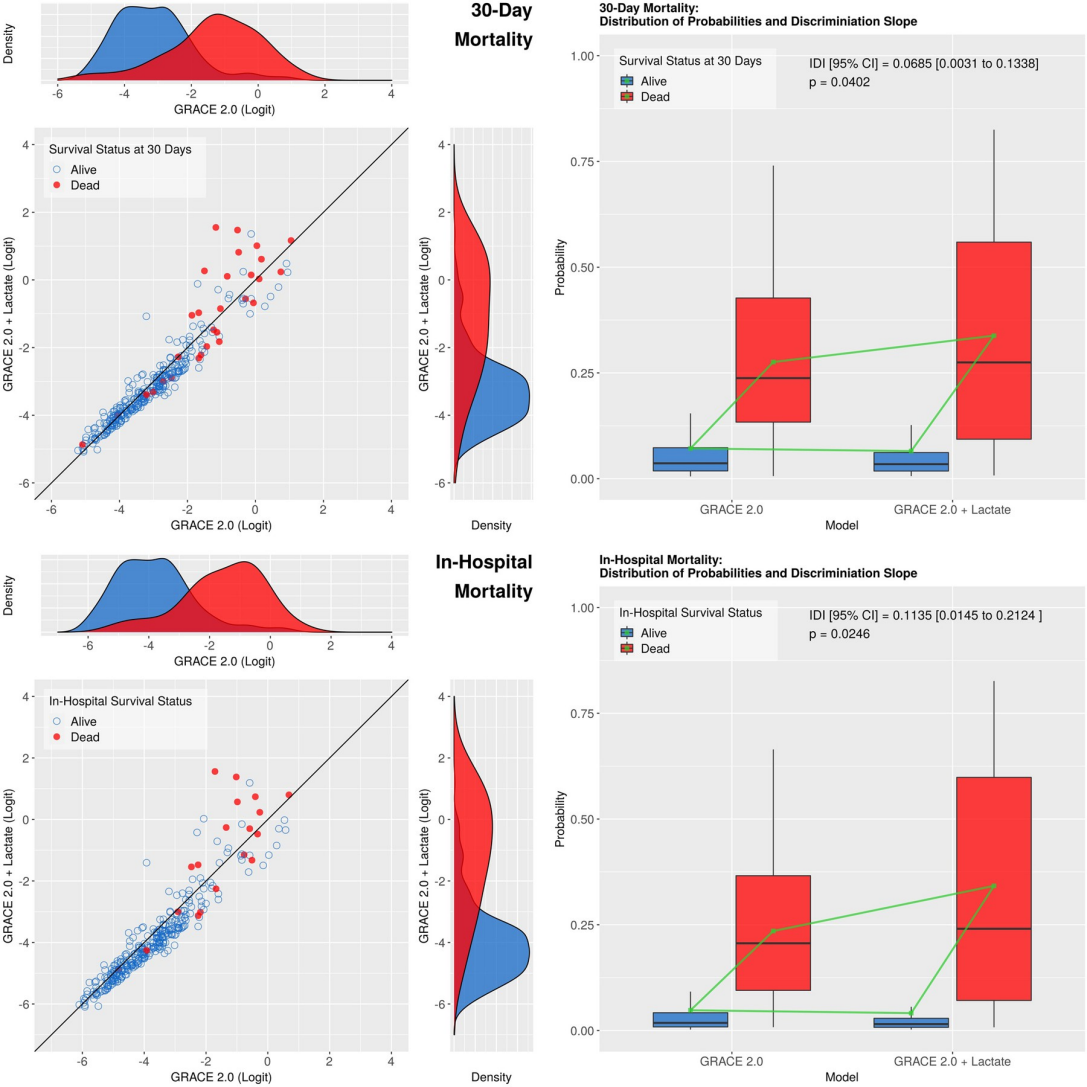
Exploratory analysis: Scatter / density (left panels) and box-and-whisker (right panels) plots for 30-day (upper panels) and in-hospital (lower panels) mortality. The left panels show combined scatter and density plots of the two models according to survival status. The diagonal line represents identical predictive ability. With the inclusion of lactate, the probability of dying within 30 days / during hospital stay was shifted downwards in most survivors (blue circles), whereas the majority of non-survivors (red dots) were shifted towards higher risk. The change in density plots also suggest an increased discriminatory power of the expanded models.

We used 30-day mortality as dependent variable as our primary outcome measure. Compared with the base model, the addition of lactate improved the model’s performance as assessed by both likelihood ratio test (LR Chi-square = 8.7967, p = 0.0030) and integrated discrimination improvement (IDI [95% Confidence Interval (CI)]: 0.0685 [0.0031 to 0.1338], p = 0.0402), suggesting that the expanded model may have better predictive ability than the GRACE 2.0 score (Fig 3, upper panel). The c-statistic was 0.8485 for the base and 0.8458 for the expanded model which were statistically not different (bootstrap test for two correlated ROC curves: p = 0.7506). The variance inflation factor was 1.1203, indicating lack of collinearity, i.e. the measured lactate values were independent of the calculated GRACE 2.0 scores.

Right panels: In the box-and-whisker plots according to survival status, the boxes represent the median and interquartile range (IQR), whereas the whiskers extend to the most extreme data point which is no more than 1.5 times the IQR from the box. The difference of mean probabilities (green squares) between non-survivors and survivors is known as discrimination slope, whereas the difference of discrimination slopes is defined as the integrated discrimination improvement (IDI).

### Secondary outcome measures

Similarly to the results with 30-day mortality, using the less exact in-hospital mortality as dependent variable, both likelihood ratio test and IDI revealed better model performance (LR test: Chi-square = 11.4213, p = 0.0007; IDI [95% CI]: 0.1135 [0.0145 to 0.2124], p = 0.0246, Fig 3, lower panel). In contrast, the c-statistic did not show a significant change in discrimination being 0.8805 and 0.8892 for the GRACE 2.0 and GRACE 2.0 plus lactate model, respectively, p = 0.3956. There was no sign of collinearity as the VIF was 1.0743.

For time- to-event analysis of the 180-day data Cox modeling was applied. Again, the likelihood ratio test demonstrated a statistically significant improvement (Chi-square = 5.9146, p = 0.0150). Nonetheless, with this relatively small sample size, the change in the discrimination slope at 180 days was not statistically relevant: IDI [95% CI]: 0.0350 [-0.0030 to 0.1090], p = 0.0730. Also, comparison of the two correlated ROC curves did not show any increase in discriminatory power with c-statistics of 0.8151 and 0.8111, for the base and expanded models, respectively, p = 0.1809. No signal of collinearity could be observed: VIF = 1.1051.

### Internal validation

**Fig 4:**
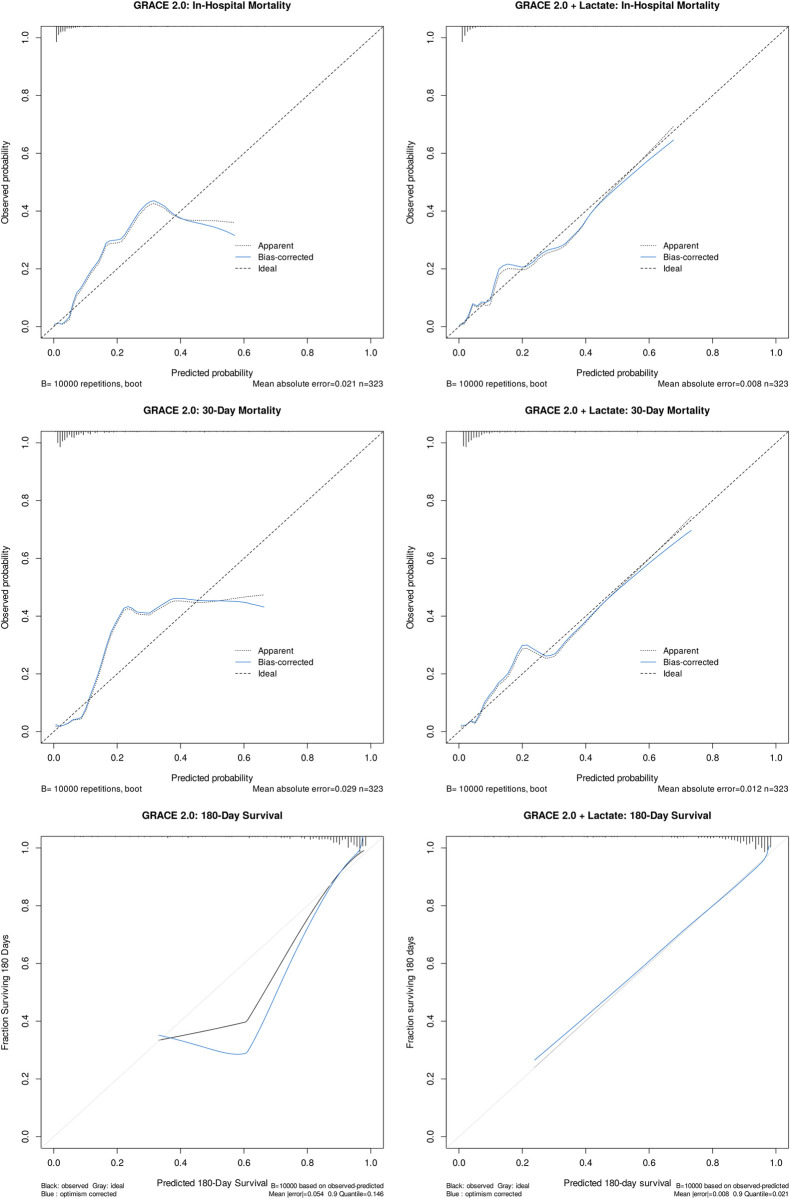
Calibration plot of the apparent and optimism-corrected models. Calibration curves were created using a smoother on the scatter plot of expected versus observed risks. Differences between predicted and observed event rates were smaller in the expanded models in both apparent and optimism-corrected models, as shown by the decreased mean absolute error. The rug plots across the top of the figures show distribution of predicted risk. Also, the le Cessie-van Houwelingen-Copas-Hosmer test for global goodness of fit demonstrated better model fit in all apparent expanded logistic regression models (Table 3).

Internal validation of the models’ discrimination and calibration was carried out by bootstrap resampling using 10000 replicates. C-statistic, calibration intercept and slope, and mean absolute error were calculated. The results are presented in Table 3. Graphical evaluation of the calibration of the apparent and optimism-corrected models is shown in Fig 4. Compared with the base models, calibration of the expanded models got better in all analyses as it is suggested by the visual analysis and the decreased mean absolute error.

**Table 3:** Internal validation.

Outcome Measure	GRACE 2.0	GRACE 2.0 + Lactate
**In-hospital mortality (19 / 323 = 5.88%)**		
*Discrimination*		
Apparent c-statistic	0.8805	0.8892
Optimism-corrected c-statistic*	0.8804	0.8865
*Calibration*		
Intercept*	0.0497	-0.0320
Slope*	1.0026	0.9658
Mean absolute error*	0.0210	0.0080
le Cessie-van Houwelingen-Copas-Hosmer test	0.0855	0.9871
**30-day mortality (29 / 323 = 8.98%)**		
*Discrimination*		
Apparent c-statistic	0.8485	0.8458
Optimism-corrected c-statistic*	0.8481	0.8429
*Calibration*		
Intercept*	0.0402	-0.0163
Slope*	1.0059	0.9767
Mdisp absolute error*	0.0290	0.0120
le Cessie-van Houwelingen-Copas-Hosmer test	0.3057	0.8568
**180-day mortality (48 / 322 = 14.91%)**		
*Discrimination*		
Apparent c-statistic	0.8151	0.8111
Optimism-corrected c-statistic*	0.8146	0.8095
*Calibration*		
Intercept*	NA	NA
Slope*	0.9961	0.9691
Mean absolute error*	0.0542	0.0083

*Results are based on 10000 bootstrap resamples.

†Ideally, the calibration intercept should be zero while the calibration slope should be equal to one.

NA = not applicable.

## Discussion

### Principal findings, general considerations

According to the current guidelines of the European Society of Cardiology, all STEMI patients’ short-term risk should be assessed early for which the GRACE 2.0 risk score is recommended [3]. As in many of the risk estimation algorithms constructed for STEMI patients, heart rate and systolic blood pressure are key predictors in this model as well [1]. Though these vital parameters may be influenced by the applied medical therapy or mechanical circulatory support, there is no evidence, that this is also accompanied by an improvement in microcirculation / prognosis of the patient. Therefore, we investigated whether the admission lactate level, a known marker of microcirculatory failure, may have an added prognostic value on top of the well validated GRACE 2.0 model. We found, that admission venous lactate level and the GRACE 2.0 score may be independent and additive predictors of 30-day all-cause mortality of STEMI patients treated with primary PCI.

In our study, we used a set of statistical metrics for new biomarkers following current recommendations [8]. The widely used c-statistic, which is known to be a relatively insensitive measure for model selection, failed to show any improvement in the expanded model using any of the investigated dependent variables [6–8, 11]. Nevertheless, its use is still recommended–even though not as primary metric–when testing new biomarkers [8].

The discriminative ability of all investigated models (and the additional prognostic value of lactate) seems to be decreasing with increasing time following the index event. This is known as time-varying discrimination performance of prognostic models with predictors with time-varying accuracy. Even though we used the 6-month GRACE score for all calculations, it seems that the constructed models are more accurate in predicting death at hospital discharge than at 30 or 180 days.

### Context with previous reports

In 2010, Vermeulen et al. drew attention to the early prognostic value of lactate as an indicator for the severity of decreased systemic blood flow with correspondingly poor outcomes in STEMI patients treated with primary PCI. Half of the non-survivors with admission lactate levels above 1.8 mmol/L died within 24 hours after presentation. Nevertheless, this cut-off value was based on tertiles, rather than formal analysis. Also, patients who were on mechanical ventilation at admission following cardiopulmonary resuscitation were excluded from the analysis [12]. Meanwhile, several other studies have been published about the prognostic importance of lactate level on survival in acute coronary syndrome patients. Yet, in most of these works, lactate level was neither treated as a continuous measure, nor was potential non-linearity investigated. Instead, it was used as a binary variable (with arbitrarily set cut-off values), which implies information loss [13–15]. Attaná et al. described the role of lower lactate-clearance in higher mortality of patients with STEMI complicated by cardiogenic shock [16]. Recently, these initial results from 51 patients were confirmed by Park et al. analyzing a large, multi-center registry with 628 cardiogenic shock patients [17]. Gjesdal et al. found that blood lactate is a predictor of short-term mortality in PCI-treated patients with myocardial infarction complicated by mild to moderate heart failure even in the absence of cardiogenic shock [18]. Unlike these previous works, we analyzed an unselected cohort of STEMI patients undergoing primary PCI and lactate level was treated as a continuous variable and checked for non-linearity, thereby preserving prognostic information. Moreover, we studied lactate level not as a stand-alone variable, but rather on top of the extensively validated GRACE 2.0 score with 8 well-established predictors.

Venous sampling may be considered as a limitation of the present work. Yet, for our pilot observational study, we used data that were readily available not requiring any intervention, as venous blood gas analysis–including the measurement of lactate–is routinely performed in all newly admitted acute patients in our cardiac intensive care unit. Moreover, it has been shown that there is a correlation between arterial and central venous (sampled from the right atrium, superior vena cava, or from the pulmonary artery) lactate levels and that the concentrations are essentially equivalent [19]. Furthermore, Younger et al. even found a strong association between arterial and peripheral venous lactate levels [20]. In a systematic review, Kruse et al. published that the correlation between lactate levels in arterial and venous blood was acceptable and venous sampling should therefore be encouraged thereby minimizing the risk and inconvenience for the patient [21]. Despite the strong correlation, the agreement is not perfect, therefore caution should be used in the routine substitution of venous for arterial blood sampling as recommended by Gallagher et al. [22]. Nevertheless, the probability of arterial hyperlactatemia may be substantially reduced, if the lactate level is normal in the venous sample [22]. For the above reasons, to completely rule out this potential bias, we are planning to repeat the study with arterial blood sampling at the time of coronary angiography.

### Potential clinical applications

Though our preliminary findings should be confirmed by larger, preferably multi-center studies before introducing it into daily practice, they may have several potential clinical implications. For example, an elevated admission lactate level in a patient with a low-to-moderate risk profile based on the GRACE 2.0 score may signal a higher risk of death and may necessitate closer patient monitoring and the search for the underlying causes. The more accurate risk prediction may provide more useful information for patients or relatives and help physicians to allocate hospital resources. It may improve intra-organizational quality monitoring. It may allow a more precise risk adjustment in inter-organizational comparisons of health care providers with different case mixes. Furthermore, it may be helpful in a more exact clinical trial design identifying patients with the needed risk profile thereby increasing statistical power or reducing sample size and costs.

### Strengths and limitations

Our results are based on a prospective registry of a single high-volume institution. We analyzed data of a real-world, relatively high-risk population treated in a contemporary fashion (i.e., high rate of transradial access site, almost exclusive use of drug eluting stents, guideline-directed discharge medications). Also, we used a set of different statistical metrics following current recommendations, treated lactate level as a continuous variable and checked for its non-linearity [8].

Yet, the single-center nature of the data does not allow generalization of the findings to populations / centers of other geographic regions. Moreover, we exclusively used venous blood samples. Furthermore, the potential effect of different P2Y_12_ receptor inhibitors on mortality was not investigated. Finally, we did not study data of non ST-segment elevation acute coronary syndrome cases. Thus, our data are not applicable in this setting.

## Conclusions

Our results suggest that admission venous lactate level and the GRACE 2.0 score may be independent and additive predictors of 30-day all-cause mortality of STEMI patients treated with primary PCI. Because of the aforementioned limitations, further, preferably multi-center randomized trials with arterial blood sampling are warranted to confirm the findings of the present study.

## Supporting information

S1 Table Analysis of variance of the expanded models. (PDF)

S1 Dataset Dataset. (CSV)
