# Supplementary material for: Admission lactate level and the GRACE 2.0 score are independent and additive predictors of 30-day mortality of STEMI patients treated with primary PCI—Results of a real-world registry
Source: PLoS One. 2022 Nov 16;17(11):e0277785. doi: 10.1371/journal.pone.0277785 (PMC9668119; doi:10.1371/journal.pone.0277785)
Supplement: S1 Table — (PDF) [file pone.0277785.s001.pdf]

S1 Table. Analysis of Variance of the Expanded Models (Wald Statistic).

Outcome Measure	GRACE 2.0	Lactate	Total
**In-hospital mortality (logistic regression)**			
Chi-Square	17.00	10.46	37.04
Degree of freedom	1	1	2
p value	<0.0001	0.0012	<0.0001
**30-day mortality (logistic regression)**			
Chi-Square	21.96	8.17	43.60
Degree of Freedom	1	1	2
p value	<0.0001	0.0043	<0.0001
**180-day mortality (Cox modeling)**			
Chi-Square	55.42	7.04	82.48
Degree of freedom	1	1	2
p value	<0.0001	0.0080	<0.0001
